# Supplementary material for: Comparative Yield of Tuberculosis during Active Case Finding Using GeneXpert or Smear Microscopy for Diagnostic Testing in Nepal: A Cross-Sectional Study
Source: Trop Med Infect Dis. 2021 Apr 14;6(2):50. doi: 10.3390/tropicalmed6020050 (PMC8167510; doi:10.3390/tropicalmed6020050)
Supplement: Supplementary file 1 [file tropicalmed-06-00050-s001.pdf]

Table S1. District wise case notification rate (CNR) of bacteriological positive in the study districts.

Province	Implementation districts	Population 2011	CNR B+ 2015/2016
Province 5 (Lumbini)	Kapilvastu	631159	98.9
	Pyuthan	237212	113.8
	Bardiya	459535	120.8
	Arghakhanchi	201055	128.3
	Gulmi	266635	110.6
Province 6 (Karnali)	Salyan	260990	91.6
Province 7 (Sudurpaschim)	Doti	213473	68.4
	Achham	276198	63.7
Total		2546257	
Province	Control districts	Population[1] 2011	CNR B+ [2] 2015/2016
Province 5 (Lumbini)	Nawalparasi	695484	107.5
	Palpa	254203	140.8
Province 6 (Karnali)	Banke	561497	158.1
Province 7 (Sudurpaschim)	Bajhang	211653	82.7
	Bajura	147526	67.8
	Total	1870363	

[1] Unpublished data (2016). National Tuberculosis Control Center

[2] Annual Report (2016). National Tuberculosis Program

Table S2. Age and sex wise category of people diagnosed with TB.

	Male (%)	Female (%)	Total (%)
0–4	0 (0)	0 (0)	0 (0)
5–14	5 (0.7)	7 (2.1)	12 (1.1)
15–24	86 (11.4)	50 (14.7)	136 (12.5)
25–34	103 (13.7)	47 (13.8)	150 (13.7)
35–44	88 (11.7)	58 (17.1)	146 (13.4)
45–54	129 (17.2)	62 (18.2)	191 (17.5)
55–64	152 (20.2)	75 (22.1)	227 (20.8)
≥ 65	179 (23.8)	51 (15.0)	230 (21.1)
Total number of TB diagnosed B+ TB (SS+ Xpert)	752 (100)	340 (100)	1092 (100)
